# Recombinant lipidated Zika virus envelope protein domain III elicits durable neutralizing antibody responses against Zika virus in mice

**DOI:** 10.1186/s12929-020-00646-x

**Published:** 2020-04-14

**Authors:** Mei-Yu Chen, Kit Man Chai, Chen-Yi Chiang, Chiao-Chieh Wu, Guann-Yi Yu, Shih-Jen Liu, Hsin-Wei Chen

**Affiliations:** 1 National Institute of Infectious Diseases and Vaccinology, National Health Research Institutes, Miaoli, Taiwan; 2 Graduate Institute of Biomedical Sciences, China Medical University, Taichung, Taiwan; 3 Graduate Institute of Medicine, College of Medicine, Kaohsiung Medical University, Kaohsiung, Taiwan

**Keywords:** Envelope protein domain III, Neutralizing antibody, Recombinant lipoprotein, Vaccine, Zika virus

## Abstract

**Background:**

The emergence of Zika virus (ZV) in tropical and subtropical areas of the world has created an urgent need for vaccines against ZV. However, approved vaccines that prevent ZV infection are not available. To develop an effective vaccine against ZV infection, a lipidated form of ZV envelope protein domain III that possesses an intrinsic adjuvant property was rationally designed. Our goal was to examine the immunogenicity of recombinant lipidated ZV envelope protein domain III (rLZE3) and evaluate its potential as a vaccine candidate against ZV.

**Methods:**

Recombinant ZV envelope protein domain III (rZE3) and rLZE3 were prepared with an *Escherichia coli*-based system. Dendritic cell surface marker expression and cytokine production upon stimulation were analyzed to evaluate the function of rLZE3. Neutralizing antibody capacities were evaluated using focus reduction neutralization tests after immunization. To investigate the protective immunity in immunized mice, serum samples collected from immunized mice were adoptively transferred into AG129 mice, and then viremia levels and survival times were examined after ZV challenge.

**Results:**

rLZE3 alone but not rZE3 alone efficiently activated dendritic cells in vitro and was taken up by dendritic cells in vivo. Immunization of C57BL/6 mice with rLZE3 alone (without exogenous adjuvant) could induce ZV-specific neutralizing antibody responses. Furthermore, serum samples obtained from rLZE3-immunized mice provided protection as indicated by a reduction in viremia levels and prolongation of survival times after ZV challenge.

**Conclusion:**

These results indicate that rLZE3 is an excellent vaccine candidate and has great potential that should be evaluated in further preclinical studies.

## Background

Zika virus (ZV) belongs to the genus Flavivirus of the family Flaviviridae. It can be spread by bites from virus-infected mosquitoes, similar to dengue virus and Japanese encephalitis virus in the same family. In recent years, the outbreak of ZV in tropical and subtropical regions has become a major public health issue [1, 2]. Infection of ZV results in a self-limiting mild illness characterized by rash, fever, conjunctivitis, arthralgia, and arthritis [3]. Recently, evidence has indicated that ZV infection can be correlated with the development of Guillain-Barré syndrome [4]. In addition, the isolation of ZV from the fetal brain and epidemiological information provide strong evidence that ZV infection is associated with microcephaly [5, 6]. At present, there are no specific curative treatments for ZV infection or approved vaccines to prevent ZV infection. Therefore, it is very important to develop a therapeutic approach or vaccine for ZV infection.

The emergence of ZV in different regions of the world has created an urgent need for vaccines against ZV [7, 8]. ZV is in the same viral family as dengue virus. It has been shown that the structure of mature ZV is arranged in a herringbone pattern with 90 antiparallel envelope dimers [9, 10], similar to the structure of dengue virus [11, 12]. The dengue virus envelope protein contains three domains. This viral structural protein plays a key role in viral entry. Envelope protein domain III (E3) of dengue virus contains an immunoglobulin-like fold and contributes to viral attachment [13, 14]. Our previous studies have shown that E3 of dengue virus is a potential dengue vaccine candidate [15–23]. It is very likely that antibodies against ZV E3 can neutralize ZV infection. Recently, ZV-derived E3 [24, 25], ZV-derived E3 displayed on immunogenic virus-like particles [26, 27], or ZV-derived E3 fused with the Fc region of human IgG [28] was found to induce neutralizing immune responses against ZV when formulated with adjuvants. The neutralizing immunogenicity on neutralizing epitopes was further enhanced by masking of a non-neutralizing epitope surrounding residue 375 of ZV E3 [29]. Characterization of the human immune response to ZV infection revealed that the highly cross-reactive antibodies to envelope protein domain I/II induced by ZV or DV infection were poorly neutralizing but potently enhancing may pose a risk for heterologous ADE [30]. Thus, ZV E3 is a potential target for ZV vaccine development.

Modern subunit vaccines comprise two critical components, an antigen and an immunostimulator. It has been shown that both synthetic lipopeptides and bacterial-derived lipoproteins are able to stimulate antigen-presenting cells through the toll-like receptor signaling pathway and thus enhance immune responses [31–33]. According to these findings, we developed a technique for producing recombinant lipoproteins with high yields and applied this technology to the development of recombinant protein-based vaccines with superior immunogenicity [34]. In this study, we produced recombinant lipidated ZV E3 (rLZE3) and evaluated the potential of rLZE3 as a vaccine candidate against ZV. We demonstrated that rLZE3 alone stimulated durable neutralizing antibody responses and produced protective effects. Our results provide valuable evidence to move the rLZE3 vaccine candidate into clinical studies in the future.

## Methods

### Cloning and expression of rZE3 and rLZE3

The DNA sequence of ZV E3 was synthesized (Purigo Biotechnology Co., Taipei, Taiwan) using *Escherichia coli* codon usage according to the amino acid sequence of ZV E3 (GenBank Acc.No. AMC13911). To construct the plasmid pZE3 for rZE3 expression, a forward primer, 5′-ACTGCGCATATGaaaggcgtgagc-3′ (the NdeI site is underlined), and a reverse primer, 5′-TCATGAATCTCGAGggtgctgccgctg-3′ (the XhoI site is underlined), were used to clone the rZE3 sequence into the NdeI and XhoI sites of the plasmid pET-22b(+) (Novagen, Madison, WI). A hexahistidine tag (His-tag) was added to the C-terminus of rZE3. For expression of rZE3, pZE3 was transformed into *E. coli* BL21 (Invitrogen, Carlsbad, CA). After transformation, the *E. coli* were cultured at 37 °C overnight. To scale up protein production, 20 ml of the overnight culture was added to 1 L of medium in a 2-L shaker flask and incubated at 37 °C for 4 h. When cultured to OD_600_ = 0.6, isopropylthiogalactoside (IPTG; 1 mM) was added, followed by an incubation for 20 h at 20 °C to induce protein expression. The D1 domain and lipid signal peptide of the lipoprotein Ag473 [34] were cloned into the NdeI and BamHI sites of the expression vector pET-22b(+) to obtain the plasmid pLipo as previously described [35]. To construct the plasmid pLZE3 for rLZE3 expression, a forward primer, 5′- GAAGATCTaaaggcgtgagctatagcct-3′ (the Bg1II site is underlined), and a reverse primer, 5′- TCATGAATCTCGAGggtgctgccgctg-3′ (the XhoI site is underlined), were used to clone the rZE3 sequence into the Bg1II and XhoI sites of the pLipo plasmid to obtain pLZE3. The C-terminus of rLZE3 contained a His-tag. For expression of rLZE3, pLZE3 was transformed into *E. coli* C43 (Lucigen, Middleton, WI). The other steps were the same as those performed for rZE3 expression.

### Production of rZE3 and rLZE3

Cells were harvested and then disrupted in a French press (Constant Systems, Daventry, UK) at 27 Kpsi in a homogenization buffer [20 mM Tris (pH 8.0), 50 mM sucrose, 500 mM NaCl and 10% glycerol]. The cell lysate was clarified by centrifugation (80,000Xg for 40 min) as previously described [35]. The majority of rZE3 was present in the inclusion bodies. rZE3 was extracted with an extraction buffer [0.02 M Tris (pH 8.0), 0.05 M sucrose, 0.5 M NaCl, 10% glycerol and 3 M GuHCl]. For purification of rZE3, the solubilized portion was loaded onto immobilized metal affinity chromatography (IMAC) columns (QIAgen, Hilden, Germany). The eluate from the IMAC column was further refined using an anion exchange column (Ni-NTA super flow; slurry). To eliminate endotoxin, the refined fraction was passed through an E membrane (Pall Co., USA). The levels of endotoxin in the purified rZE3 fraction were evaluated by a Limulus amebocyte lysate (LAL) assay (Associates of Cape Cod, Inc., Cape Cod, MA). The residual endotoxin concentration was less than 10 EU/mg. After elimination of endotoxin, rZE3 was dialyzed against 0.01 M dibasic sodium phosphate, lyophilized and stored at − 20 °C. Fractions collected throughout this process were evaluated by SDS-PAGE and immunoblotting with an anti-His-tag antibody. For preparation of rLZE3, the target protein was extracted with an extraction buffer [0.02 M Tris (pH 8.0), 0.05 M sucrose, 0.5 M NaCl, 10% glycerol, 1% TritonX-100, and 3 M GuHCl]. rLZE3 was dialyzed against 0.01 M dibasic sodium phosphate/0.01 M mannitol/3 mg/ml sucrose. The other processes were the same as those used for rZE3 purification.

### Identification of the lipid moiety in rLZE3

After digestion of rLZE3 with trypsin (Sigma, St. Louis, MO), the digestion mixture was further refined with a ZipTip (Millipore, Massachusetts). The ZipTip-refined trypsin-digested fragments (1 μL) were mixed with 1 mL of an α-cyano-4-hydroxycinnamic acid saturated solution in acetonitrile/0.1% trifluoroacetic acid (1:3 vol:vol). The mixture (1 μL) was placed on the target plate of a MALDI micro MX mass spectrometer (Waters, Manchester, UK) for analysis as previously described [35].

### Effect of rLZE3 on dendritic cell activation

Bone marrow was harvested from the femurs and tibiae of C57BL/6 mice (*n* = 2–3 in each independent experiment). After vigorous pipetting, red blood cells were removed using a lysis buffer. The isolated bone marrow cells (2–5 X 10^5^ cells/mL) were cultured in RPMI-1640 medium supplemented with β-mercaptoethanol (0.05 mM), L-glutamine (2 mM), HEPES (20 mM), penicillin/streptomycin (100 units/mL), and heat-inactivated fetal bovine serum (10%, v/v) at 37 °C in a 5% CO_2_ atmosphere. Granulocyte macrophage colony stimulating factor (200 units/mL) was added to the cultures on days 0 and 3. On days 6–7, the cultured cells were harvested and seeded in 24-well plates (1 X 10^6^ cells/mL/well) and stimulated with rZE3 or rLZE3 (100 nM). After stimulation for 20 h, the levels of TNF-α, IL-6, and IL-12p40 in the supernatants were examined using specific cytokine ELISA kits (eBioscience, San Diego, CA). The expression of CD11c, CD40, and CD80 on the cell surface was determined by staining with anti-CD11c, anti-CD40, and anti-CD80 monoclonal antibodies for evaluation by flow cytometry (FACSCalibur, BD Biosciences). Data acquisition was performed by using CellQuest Pro software, and data were analyzed using FACS 3 software to evaluate gated CD11c^+^ cell populations.

### Antigen uptake by dendritic cells

To evaluate internalization of antigens in the vaccinated mice in vivo, rZE3 and rLZE3 were labeled by Alexa Fluor 647 labelling kit (Thermo Fisher Scientific, MA). Groups (4 mice/group) of C57BL/6 mice (6–8 weeks of age) were treated with footpad injections of 100 μg alexa647-labeled rZE3 or rLZE3 (100 μg into one side footpad). Single suspension lymphocytes were prepared from draining lymph nodes which derived from mice 24 h after injection. The LIVE/DEAD fixable dead cell stain kits (Thermo Fisher Scientific, MA) was used to evaluate the viability of lymphocytes by flow cytometry. B cells, T cells, NK cells, and neutrophils were stained with FITC-CD19, CD3e, NK1.1, and Ly6G (1A8) antibody. Dendritic cells were defined with PerCP-Cy5.5 conjugated-MHCII and BV421-CD11c. Staining antibodies were obtained from Biolegend. Lymphocytes in draining lymph node was analyzed by flow cytometry.

### Experimental mice and immunizations

C57BL/6 mice were obtained from the National Laboratory Animal Breeding and Research Center (Taipei, Taiwan). AG129 mice were bred at the Laboratory Animal Center of the National Health Research Institutes. All the mice were housed at the Laboratory Animal Center of the National Health Research Institutes. Animals (6–8 weeks old mice) were immunized with vaccine candidates via subcutaneous injection at the indicated doses. Mice (4–5 mice/group) received 2 vaccinations at a 2-week interval with the same regimen. Serum samples were collected by tail bleeding each mouse at different time points as indicated.

### Measurement of antibody titers

Anti-rZE3 IgG titers in the serum were determined by titration as previously described with some modifications [21]. Serum samples were prepared in 3-fold serial dilutions (starting at 1:30) and then added to rZE3-coated 96-well plates. Bound IgG was identified by a goat anti-mouse IgG Fc antibody conjugated with horseradish peroxidase. After washing with PBS and the addition of a 3,3′,5,5′-tetramethylbenzidine substrate, the absorbance at 450 nm was determined using an ELISA reader. The serum dilution that produced an OD value of 0.3 was defined as the ELISA endpoint titer. Titers were calculated from the titration curve by interpolation unless the OD value was less than 0.3 at the starting dilution (1:30).

### Focus-forming assays

Plasma samples from challenged mice were diluted using 10-fold serial dilutions (starting at 1:10). Virus titers were determined as previously described with some modifications [21]. Diluted plasma was allowed to infect a monolayer of Vero cells in 24-well plates at 37 °C. After 3 h of incubation, an overlay of medium containing 2.5% fetal bovine serum and 0.8% methylcellulose in DMEM was added at the conclusion of the infection period. The infected monolayer was incubated at 37 °C for 55 h, and then the overlay medium was removed. After washing with phosphate-buffered saline (PBS), the cells were fixed for 15 min in 3.7% formaldehyde/PBS, permeabilized with 0.1% nonidet P40/PBS for 15 min and blocked with 3% bovine serum albumin/PBS for 15 min. ZV-infected Vero cells were identified by the HB122 anti-ZV antibody. After washing with PBS, antibody-bound cells were identified using a horseradish peroxidase-conjugated goat anti-mouse IgG (H + L) antibody. The infected cells were visualized using TMB.

### Focus reduction neutralization tests (FRNT)

Heat-inactivated serum samples were prepared in 2-fold serial dilutions (starting at 1:8). Neutralizing antibody titers were determined as previously described with some modifications [21]. ZV was incubated with the serum samples at 4 °C overnight in a final volume of 200 μL. The mixture was added to monolayers of Vero cells in 24-well plates. Focus-forming units (FFUs) were determined by focus-forming assays. The FRNT_50_ neutralizing antibody titer was defined as the highest dilution that resulted in a 50% reduction in FFUs compared to the FFUs of negative control samples that contained virus alone. Any neutralizing antibody titers below 8 were designated as 4 for calculation purposes.

### Animal challenge

AG129 mice (9–11 weeks old mice) were intraperitoneally injected with 0.4 mL of serum from immunized C57BL/6 mice. After 6 h, the AG129 mice were intraperitoneally injected with 50 FFUs of ZV (PRVABC59) in 0.2 mL of PBS. Blood samples were obtained at 3 days post-ZV challenge. The blood samples (0.2 mL) were instantly mixed with prechilled 3.8% sodium citrate (20 μL). The virus titers in the plasma were determined using focus-forming assays with Vero cells. If the virus titers were less than 2.0 log_10_FFU/mL (detection limit of the assay), a value of 1.0 was assigned for calculation purposes.

### Data analysis

Values are presented as the mean ± SEM. The Kruskal-Wallis test with Dunn’s multiple comparison test was performed to compare differences among more than two groups. GraphPad Prism software version 5.02 (GraphPad Software, San Diego, CA) was used for statistical analysis. Differences with *p* < 0.05 were considered statistically significant.

## Results

### Preparation and characterization of recombinant Zika virus envelope protein domain III proteins

**Fig. 1:**
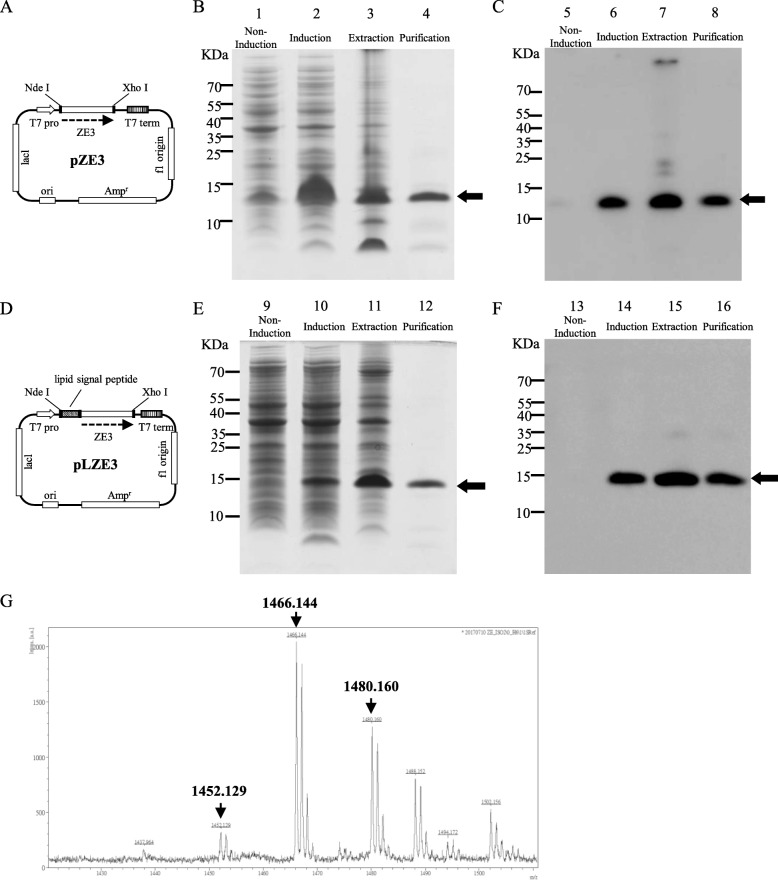
Production and purification of recombinant Zika virus envelope protein domain III (rZE3) and recombinant lipidated Zika virus envelope protein domain III (rLZE3). The plasmid maps of pZE3 (**a**) and pLZE3 (**d**) for the production of rZE3 and rLZE3, respectively. The purification of rZE3 (**b**, **c**) and rLZE3 (**e**, **f**) was monitored by 10% reducing Tricine-SDS-PAGE followed by Coomassie Blue staining and immunoblotting with anti-His-tag antibodies. rZE3 and rLZE3 were expressed in the *E. coli* strains BL21 (DE3) and C43 (DE3), respectively. Lanes 1, 5, 9, and 13: protein expression without IPTG induction; lanes 2, 6, 10, and 14: protein expression after IPTG induction; lanes 3 and 7: extraction of rZE3 from inclusion bodies; lanes 11 and 15: soluble fraction of rLZE3; and lanes 4, 8, 12, and 16: purified proteins. Lanes 5–8 and lanes 13–16 show the induction and purification processes for rZE3 and rLZE3, respectively, evaluated by immunoblotting. The arrows show the electrophoretic positions of rZE3 or rLZE3. **g** Mass spectrum analysis of rLZE3. The N-terminus of the rLZE3 fragments was obtained by trypsin digestion and further examined with a WatersR MALDI micro MX™ mass spectrometer. MALDI-TOF MS spectra revealed lipid peptide signals with three m/z value peaks of 1452.129, 1466.144, and 1480.160

We constructed the plasmids pZE3 (Fig. 1a) and pLZE3 (Fig. 1d) to produce rZE3 and rLZE3, respectively. Conditions for the preparation of rZE3 and rLZE3 were tested, and purified rZE3 (Fig. 1b and c) and rLZE3 (Fig. 1e and f) were obtained. The concentrations of residual LPS in the rZE3 and rLZE3 preparations were less than 10 EU/mg after removal of LPS. Next, the exact mass of trypsin-digested rLZE3 N-terminal fragments was examined. Three major peaks were identified with m/z values of 1452, 1466, and 1480 (Fig. 1g). These peaks are considered to be lipidation signatures that have been confirmed in other recombinant lipidated proteins [16, 18, 19, 22].

### Functional assessment of recombinant lipidated Zika virus envelope protein domain III

**Fig. 2:**
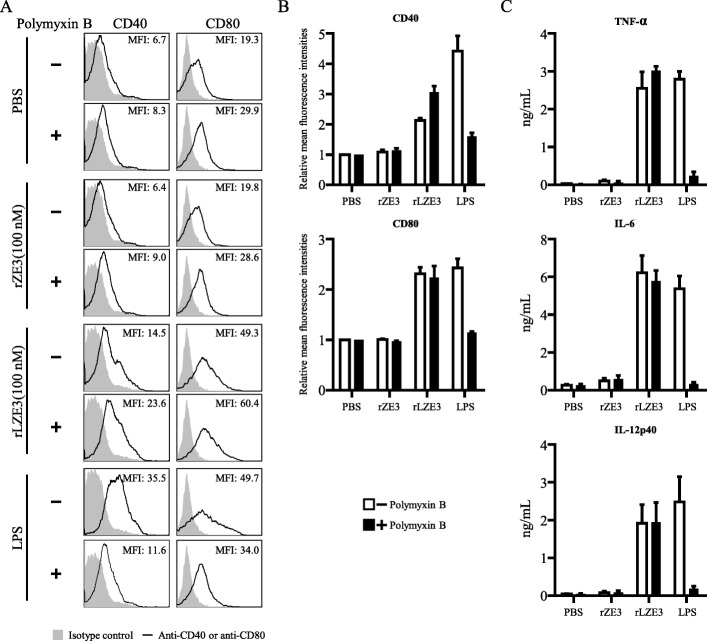
Effect of rLZE3 on the activation of bone marrow-derived dendritic cells. Bone marrow-derived dendritic cells were stimulated with rZE3 or rLZE3 (100 nM) with or without polymyxin B (25 μg/mL). PBS and lipopolysaccharide stimulations were used as controls. **a** Dendritic cells were harvested after 20 h of stimulation. CD40 and CD80 expression levels in a CD11c^+^ gated population were evaluated by flow cytometry. A representative experiment is shown. **b** The mean fluorescence intensity (MFI) of cells stimulated with PBS was defined as the basal expression level. Relative MFIs are shown. The data represent the mean ± SE of the mean from three independent experiments. **c** TNF-α, IL-6 and IL-12p40 levels in the supernatants after 20 h of stimulation were evaluated by ELISA kits. The data represent the mean ± SE of the mean from three independent experiments

Recombinant lipidated proteins produced by bacteria are able to stimulate antigen-presenting cells through toll-like receptor signaling pathways. The functionality of the rLZE3 lipid moiety was evaluated by stimulating bone marrow-derived dendritic cells with rZE3 or rLZE3. The expression levels of CD40 and CD80 on the bone marrow-derived dendritic cells were examined by flow cytometry. rLZE3 increased the CD40 and CD80 expression levels, while rZE3 (without lipidation) did not enhance CD40 and CD80 expression (Fig. 2a). In addition, we added polymyxin B to the stimulation to eliminate the effect of any minor residual endotoxin remaining after rZE3 or rLZE3 purification. It was evident that there were no substantial reduction effects of polymyxin B on stimulation with rZE3 or rLZE3. In contrast, adding polymyxin B abolished enhancing effects of LPS. The mean fluorescence intensity of bone marrow-derived dendritic cells cultured without stimulation (PBS) was defined as the basal expression level. The relative mean fluorescence intensities from three independent experiments are summarized in Fig. 2b. These results indicate that the lipid moiety of rLZE3 leads to enhancement of the expression of CD40 and CD80. Furthermore, rLZE3 was able to stimulate the production of TNF-α, IL-6 and IL-12p40 by bone marrow-derived dendritic cells. In the presence of polymyxin B, the production of cytokines was not eliminated. In contrast, rZE3 (the counterpart of rLZE3 that lacks lipidation) was unable to enhance cytokine production (Fig. 2c). These results support the conclusion that rLZE3 stimulates bone marrow-derived dendritic cells.

**Fig. 3:**
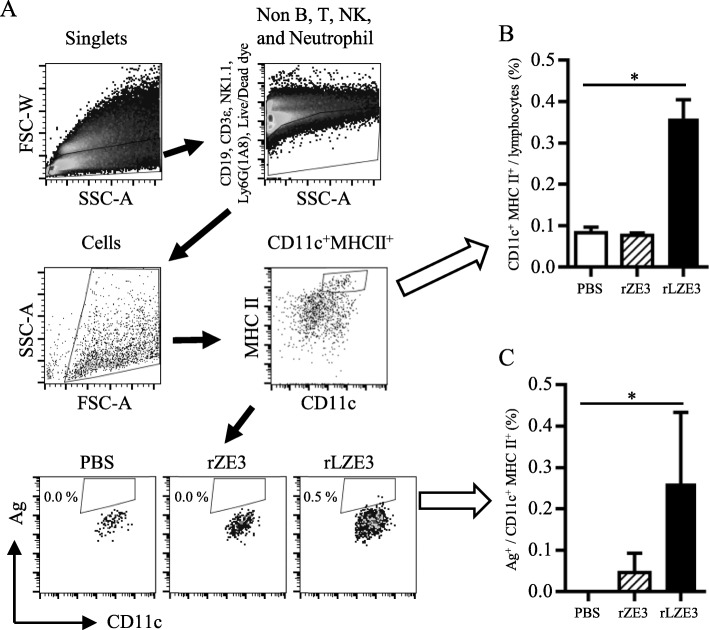
Enhancement of antigen uptake by dendritic cells and elevation of the dendritic cell frequency in the draining lymph nodes mediated by the injection of LZE3. **a** Gating strategy for the dendritic cell population in the draining lymph nodes. Alexa Fluor 647-labeled rZE3 or rLZE3 was injected into the hind foot pads (100 μg/foot pad) of C57BL/6 mice. Mice injected with PBS were used as controls. Cells were harvested 24 h after injection. Single cells were gated by FSC-W/SSC-A. Dead cells, B cells, T cells, NK cells, and neutrophils were excluded from the analysis by staining with a Live/Dead® fixable dead cell stain dye and anti-CD19, anti-CD3e, anti-NK1.1, and anti-Ly6G (1A8) antibodies. The frequencies of CD11c^+^MHC II^+^ cells (**b**) and antigen-labeled CD11c^+^MHC II^+^ cells (**c**) were further analyzed. The results shown are from one of two representative experiments. Statistical significance was determined using the Kruskal-Wallis test with Dunn’s multiple comparison test. *p < 0.05

To examine whether rLZE3 enhances antigen capture by dendritic cells, PBS or Alexa Fluor 647-conjugated rZE3 or rLZE3 was injected into mouse foot pads. The frequencies of CD11c^+^MHC II^+^ cells and antigen-containing CD11c^+^MHC II^+^ cells in the inguinal lymph nodes were analyzed by flow cytometry at 24 h after injection. The gating strategy and representative results are shown in Fig. 3a. Injection of rLZE3 elevated the frequency of CD11c^+^MHC II^+^ cells in the inguinal lymph nodes compared with injection of rZE3 or PBS (Fig. 3b). Furthermore, the frequency of antigen-containing CD11c^+^MHC II^+^ cells also increased in the mice injected with rLZE3 (Fig. 3c). These results suggest that the injection of rLZE3 can increase the dendritic cell frequency in draining lymph nodes and enhance antigen uptake by dendritic cells.

### Assessment of the antibody response to Zika virus envelope protein domain III in mice

**Fig. 4:**
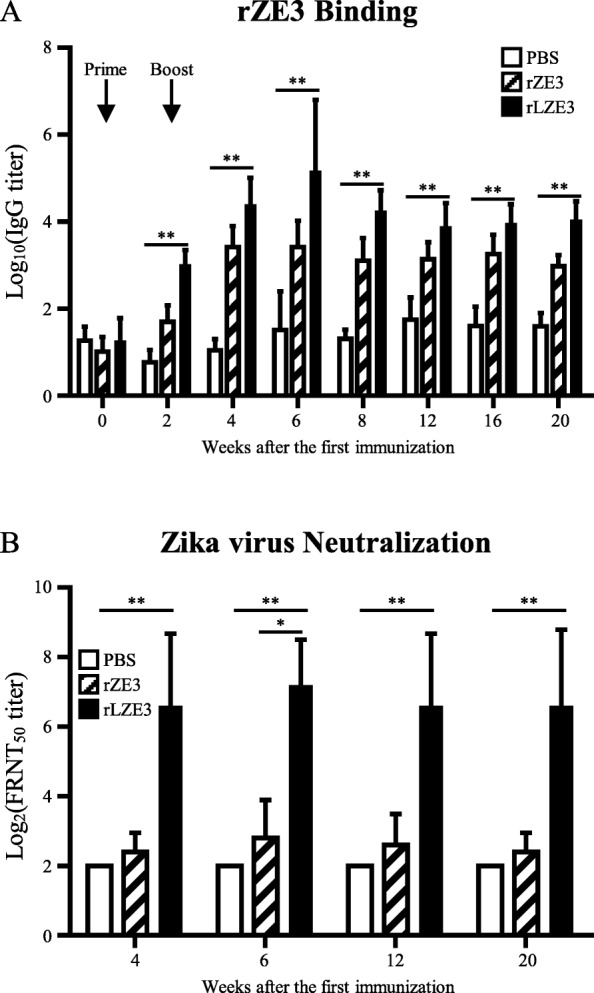
Antibody responses induced by rLZE3. C57BL/6 mice (*n* = 5/group) received two vaccinations with PBS, rZE3, or rLZE3 (10 μg per dose) via the subcutaneous route at a two-week interval. Serum samples were collected from vaccinated mice at the indicated time points after the first vaccination. **a** The titers of anti-rZE3 IgG antibodies were determined by ELISA. Serum samples were collected before vaccination (week 0) and used to determine basal levels for comparison. **b** The Zika virus-neutralizing capacity of the serum samples was determined by FRNT. The neutralizing antibody titer was defined as the reciprocal of the highest dilution that resulted in a 50% reduction in FFUs compared to the FFUs of control samples containing the virus alone. The data represent the mean ± SE of the mean. Statistical significance was determined using the Kruskal-Wallis test with Dunn’s multiple comparison test. *p < 0.05; **p < 0.01

The immunogenicity of purified rZE3 and rLZE3 was evaluated in mice. Groups of C57BL/6 mice received two immunizations with PBS, rZE3 or rLZE3 (10 μg per dose) with a two-week interval between immunizations. The immunized mice were bled to collect serum at the indicated time points. We found that rLZE3 possessed high immunogenicity. Mice immunized with rLZE3 alone exhibited superior antibody responses compared with mice immunized with rZE3. In addition, antibody responses were quickly generated in mice that received one dose of rLZE3 (at 2 weeks post priming). After a booster vaccination, antibody titers were further increased and sustained over 20 weeks after the first vaccination (Fig. 4a).

Next, we evaluated the neutralizing capacity of the antibodies elicited by vaccination. As shown in Fig. 4b, mice immunized with rZE3 were unable to generate significant neutralizing antibody responses even when immunized twice. Remarkably, mice immunized with rLZE3 exhibited significant neutralizing antibody titers at 4 weeks after priming. The neutralizing antibody titers were maintained for at least 20 weeks after the initial priming immunization. These results suggest that mice immunized with rLZE3 in an exogenous adjuvant-free formulation develop long-lasting neutralizing antibody responses.

**Fig. 5:**
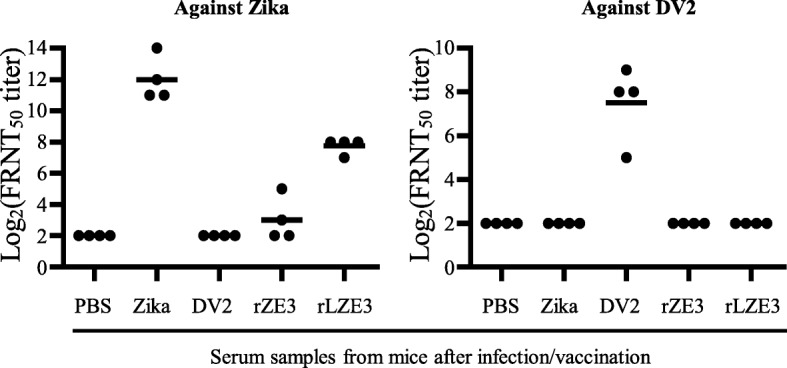
Neutralizing antibody responses induced by rLZE3 do not cross-neutralize dengue-2 virus. Groups of C57BL/6 mice (*n* = 4) were immunized subcutaneously with PBS, rZE3, or rLZE3 (10 μg per dose) twice at a two-week interval. Live Zika virus or dengue-2 virus was injected intraperitoneally in parallel. Serum samples were collected from immunized mice at 8 weeks after the first immunization. The Zika virus- or dengue-2 virus-neutralizing capacity of the serum samples was determined by FRNT. The neutralizing antibody titer was calculated as the reciprocal of the highest dilution that resulted in a 50% reduction in FFUs compared to the FFUs of control samples containing the virus alone

To examine whether the neutralizing antibodies induced by rLZE3 neutralize dengue-2 virus, we prepared serum samples from mice immunized with rZE3 or rLZE3 or infected with ZV or dengue-2 virus. We found that the serum samples obtained from the mice immunized with rLZE3 or infected with ZV could neutralize ZV but not dengue-2 virus. In contrast, the serum samples obtained from the mice infected with dengue-2 could neutralize dengue-2 virus but not ZV (Fig. 5). These results indicate that the neutralizing antibody responses induced by rLZE3 do not cross-neutralize dengue-2 virus.

### Induction of functional immunity against Zika virus in mice

**Fig. 6:**
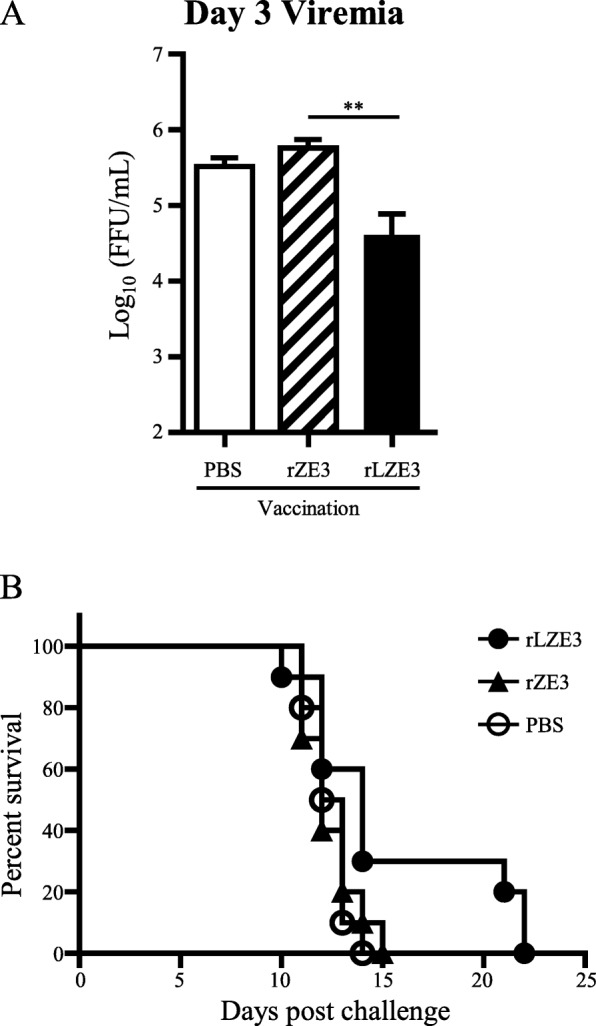
Inhibition of viremia levels and prolongation of survival time in rLZE3-immunized mice. Groups of C57BL/6 mice (*n* = 9–10/group) were immunized subcutaneously with PBS, rZE3, or rLZE3 (50 μg per dose) twice at a three-week interval. Serum samples were collected at 8–10 weeks after the first immunization. AG129 mice were intraperitoneally injected with 0.4 of mL sera from C57BL/6 mice that received different immunizations. After 6 h, the AG129 mice (*n* = 10/group) were intraperitoneally injected with 50 FFUs of Zika virus (PRVABC59) in 0.2 mL of PBS. **a** The mice were bled 3 days after being challenged. The viral titers in the plasma were evaluated by focus-forming assays using Vero cells. The plasma virus titers were logarithmically transformed before statistical analyses. Data represent the mean ± SE of the mean. Statistical significance was determined using the Kruskal-Wallis test with Dunn’s multiple comparison test. *p < 0.05. **b** The overall survival of the mice is shown

To evaluate the protective immunity in immunized mice, serum samples were collected at 6–8 weeks after the first immunization. The sera were adoptively transferred into AG129 mice. These animals were challenged with ZV 6 h after receiving the sera. The viremia levels of the mice that received the sera from rLZE3-immunized mice were lower than those of the mice that received the sera from rZE3- or PBS-immunized mice (Fig. 6a). In addition, the mice that received the rLZE3-immunized mouse sera exhibited prolonged survival times (Fig. 6b). These results suggest that rLZE3 vaccination induces neutralizing antibodies which are capable of neutralizing ZV in culture and decrease viral replication in mice.

## Discussion

In this study, we aimed to evaluate the possibility of applying rLZE3 as a potential ZV subunit vaccine candidate. We showed that rLZE3 could be readily prepared with an *E. coli*-based system (Fig. 1) and that immunization with rLZE3 could elicit durable neutralizing antibody responses in mice. Several studies have reported that ZV E3 contains epitopes recognized by strong neutralizing monoclonal antibodies [30, 36–39], suggesting that ZV E3 is a potential target that can trigger neutralizing antibody responses. Consistent with these findings, our results show that rLZE3 potently induces specific neutralizing antibodies against ZV in mice (Figs. 4b and 5). Notably, we demonstrated that passive transfer of rLZE3-immunized mouse sera reduced viremia levels (Fig. 6a) and prolonged survival times (Fig. 6b) in recipient mice challenged with ZV. Similar observations have been made in other studies [40, 41]. Collectively, these results suggest that neutralizing antibodies play a critical role in providing protective immunity against ZV infection.

In general, purified recombinant proteins are not immunogenic. Adjuvants are often required in protein-based subunit vaccine formulations to enhance the antigen-specific immune response. It has been shown that *E. coli*-produced recombinant lipidated antigens are efficiently captured by dendritic cells [35]. In addition, these recombinant lipidated antigens can also stimulate dendritic cell activation and further enhance antigen-specific immune responses [18–20, 34, 35]. In agreement with these findings, our results showed that rLZE3 but not its nonlipidated counterpart could stimulate dendritic cells to increase the expression of CD40 and CD80 (Fig. 2a and b) and enhance the production of cytokines (Fig. 2c). We further demonstrated that mice injected with rLE3 exhibited increased frequencies of not only dendritic cells in the draining lymph node but also antigen-loaded dendritic cells (Fig. 3). All of the above-mentioned features of rLZE3 conferred robust immune responses. As a consequence, rLZE3 alone, without exogenous adjuvant, induced neutralizing antibodies superior to those induced by rZE3. These results suggest that use of a recombinant lipidated antigen is a potent strategy for protein-based subunit vaccine development.

Both ZV and DV2 belong to the genus Flavivirus. Sera from rLZE3-immunized mice can bind to recombinant dengue-2 E3 (rD2E3) and slightly bind to DV2 (Additional file 1: Figure S1). However, sera from rLZE3-immunized mice did not neutralize DV2 (Fig. 5). It may increase the infection of DV2 in the rLZE3-immunized hosts. To address this issue, we further examined the capacities of sera from rZE3-, rLZE3-, ZV-, and DV2-immunized mice to mediate antibody-dependent enhancement (ADE) of DV2 infection. Our results show that ZV- and DV2-immunized sera, but not rZE3- or rLZE3-immunized sera, exhibit great ADE capacities of DV2 infection (Additional file 2: Figure S2). In line with other studies, ZE3-based vaccines formulated with adjuvant elicit neutralizing antibodies and little ADE effects to DV [24–27]. Here, we show rLZE3 with intrinsic adjuvant also induces neutralizing antibodies and reduces risk of ADE. These results indicate that rLZE3 is a potential vaccine candidate and superior to whole virion in terms of minimal risk to induce ADE for DV.

Induction of a durable immune response is a key index of a good vaccine candidate. We showed that the induced neutralizing antibodies persisted for at least 20 weeks after priming without the use of exogenous adjuvant in our formulation (Fig. 4b). These results suggest that rLZE3 is a potent vaccine candidate against ZV.

## Conclusions

In summary, our results show that rLZE3 can be robustly produced in an *E. coli*-based system and that rLZE3 alone potently induces neutralizing antibodies against ZV infection. Passive transfer of rLZE3-immunized sera reduced viremia levels and prolonged survival times in recipient mice challenged with ZV. Collectively, our results demonstrate that *E. coli*-produced rLZE3 is a potential ZV vaccine candidate worthy of further development.

## Supplementary information

**Additional file 1: Figure S1.** The capability of rLZE3 induced antibodies bind to rDE3 and dengue-2 virus. C57BL/6 mice were immunized subcutaneously with PBS (*n* = 4), rZE3 (*n* = 5), or rLZE3 (n = 5) (10 μg per dose) twice at a two-week interval. Serum samples were collected from immunized mice at 8 weeks after the first immunization. ELISA was performed by using rZE3 (A) or dengue-2 virus (B) as coating antigen. Data represent the mean ± SE of the mean.

**Additional file 2: Figure S2.** Assessment of rLZE3 induced antibodies mediate antibody-dependent enhancement for dengue-2 virus infection. C57BL/6 mice were immunized subcutaneously with PBS, rZE3, or rLZE3 (10 μg per dose) twice at a two-week interval. Live Zika virus or dengue-2 virus was injected intraperitoneally in parallel. Serum samples were collected from immunized mice at 8 weeks after the first immunization. Antibody-mediated enhancement of dengue virus infectivity was determined by flow cytometry in K562 cells. Sera were diluted via 4-fold serial dilutions (starting at 1∶4), and the sera were heatinactivated prior to testing. Serially diluted sera and virus were mixed and incubated to form immune complexes for 1 h at 37 °C. K562 cells were mixed with immune complexes (MOI  =  0.1) and then incubated for 1.5 h at 37 °C. After washing, the cells were resuspended in fresh medium and incubated for 3 days at 37 °C. Infections with and without virus were performed in parallel as controls. Cells were stained for intracellular with monoclonal anti-dengue antibodies (American Type Culture Collection, No. HB-114 for dengue-2). Antibody-labeled cells were detected with a secondary antibody conjugated to FITC. The data were acquired with CellQuest Pro software on a BD FACSCalibur flow cytometer and were analyzed with FCS Express software.

## Data Availability

The datasets analyzed in the current study are available upon reasonable request.

## Abbreviations

E3: Envelope protein domain III
FRNT: Focus reduction neutralization tests;
rLZE3: Recombinant lipidated Zika virus envelope protein domain III
rZE3: Recombinant Zika virus envelope protein domain III
ZV: Zika virus
