# Supplementary material for: Recombinant lipidated Zika virus envelope protein domain III elicits durable neutralizing antibody responses against Zika virus in mice
Source: J Biomed Sci. 2020 Apr 14;27:51. doi: 10.1186/s12929-020-00646-x (PMC7158147; doi:10.1186/s12929-020-00646-x)
Supplement: Supplementary file 1 — Additional file 1: Figure S1. The capability of rLZE3 induced antibodies bind to rDE3 and dengue-2 virus. C57BL/6 mice were immunized subcutaneously with PBS (n = 4), rZE3 (n = 5), or rLZE3 (n = 5) (10 μg per dose) twice at a two-week interval. Serum samples were collected from immunized mice at 8 weeks after the first immunization. ELISA was performed by using rZE3 (A) or dengue-2 virus (B) as coating antigen. Data represent the mean ± SE of the mean. [file 12929_2020_646_MOESM1_ESM.pdf]

Figure S1

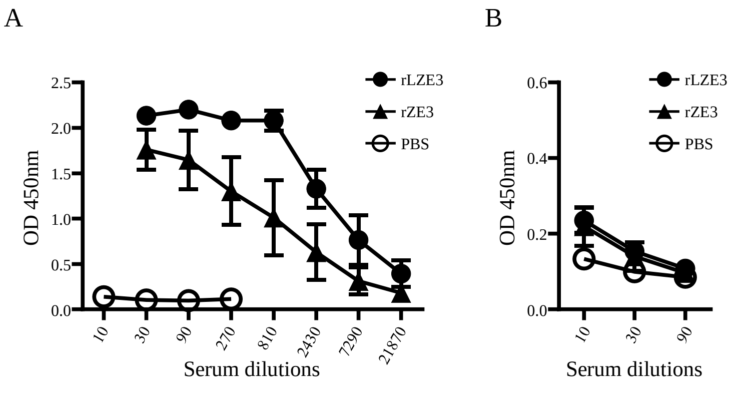

Figure S1. The capability of rLZE3 induced antibodies bind to rDE3 and dengue-2 virus. C57BL/6 mice were immunized subcutaneously with PBS (n=4), rZE3 (n=5), or rLZE3 (n=5) (10 μg per dose) twice at a two-week interval. Serum samples were collected from immunized mice at 8 weeks after the first immunization. ELISA was performed by using rZE3 (A) or dengue-2 virus (B) as coating antigen. Data represent the mean ± SE of the mean.
