# Supplementary material for: Recombinant lipidated Zika virus envelope protein domain III elicits durable neutralizing antibody responses against Zika virus in mice
Source: J Biomed Sci. 2020 Apr 14;27:51. doi: 10.1186/s12929-020-00646-x (PMC7158147; doi:10.1186/s12929-020-00646-x)
Supplement: Supplementary file 2 — Additional file 2: Figure S2. Assessment of rLZE3 induced antibodies mediate antibody-dependent enhancement for dengue-2 virus infection. C57BL/6 mice were immunized subcutaneously with PBS, rZE3, or rLZE3 (10 μg per dose) twice at a two-week interval. Live Zika virus or dengue-2 virus was injected intraperitoneally in parallel. Serum samples were collected from immunized mice at 8 weeks after the first immunization. Antibody-mediated enhancement of dengue virus infectivity was determined by flow cytometry in K562 cells. Sera were diluted via 4-fold serial dilutions (starting at 1∶4), and the sera were heatinactivated prior to testing. Serially diluted sera and virus were mixed and incubated to form immune complexes for 1 h at 37 °C. K562 cells were mixed with immune complexes (MOI = 0.1) and then incubated for 1.5 h at 37 °C. After washing, the cells were resuspended in fresh medium and incubated for 3 days at 37 °C. Infections with and without virus were performed in parallel as controls. Cells were stained for intracellular with monoclonal anti-dengue antibodies (American Type Culture Collection, No. HB-114 for dengue-2). Antibody-labeled cells were detected with a secondary antibody conjugated to FITC. The data were acquired with CellQuest Pro software on a BD FACSCalibur flow cytometer and were analyzed with FCS Express software. [file 12929_2020_646_MOESM2_ESM.pdf]

Figure S2

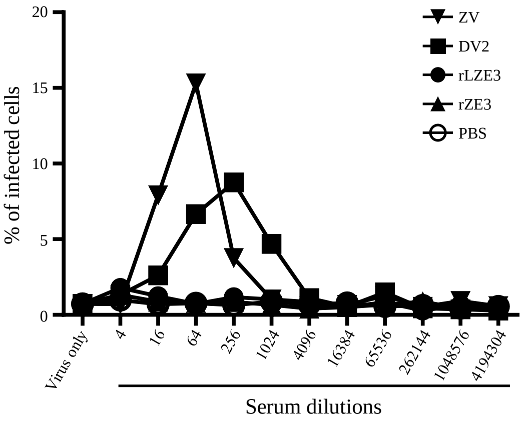

Figure S2. Assessment of rLZE3 induced antibodies mediate antibody-dependent enhancement for dengue-2 virus infection. C57BL/6 mice were immunized subcutaneously with PBS, rZE3, or rLZE3 (10 µg per dose) twice at a two-week interval. Live Zika virus or dengue-2 virus was injected intraperitoneally in parallel. Serum samples were collected from immunized mice at 8 weeks after the first immunization. Antibody-mediated enhancement of dengue virus infectivity was determined by flow cytometry in K562 cells. Sera were diluted via 4-fold serial dilutions (starting at 1:4), and the sera were heat-inactivated prior to testing. Serially diluted sera and virus were mixed and incubated to form immune complexes for 1 h at 37°C. K562 cells were mixed with immune complexes (MOI=0.1) and then incubated for 1.5 h at 37°C. After washing, the cells were resuspended in fresh medium and incubated for 3 days at 37°C. Infections with and without virus were performed in parallel as controls. Cells were stained for intracellular with monoclonal anti-dengue antibodies (American Type Culture Collection, No. HB-114 for dengue-2). Antibody-labeled cells were detected with a secondary antibody conjugated to FITC. The data were acquired with CellQuest Pro software on a BD FACSCalibur flow cytometer and were analyzed with FCS Express software.
